# Effect of *Lactobacillus brevis* CD2 containing lozenges and plaque pH and cariogenic bacteria in diabetic children: a randomised clinical trial

**DOI:** 10.1007/s00784-020-03342-0

**Published:** 2020-10-21

**Authors:** Stefano Lai, Peter Lingström, Maria Grazia Cagetti, Fabio Cocco, Gianfranco Meloni, Maria Antonietta Arrica, Guglielmo Campus

**Affiliations:** 1 Department of Biomedical Sciences-Medical School, University of Sassari, Sassari, Italy; 2 Department of Cariology, Institute of Odontology, The Sahlgrenska Academy, University of Gothenburg, Gothenburg, Sweden; 3 Department of Biomedical, Surgical and Dental Sciences, University of Milan, Milan, Italy; 4 Department of Surgery, Medical and Experimental Sciences, University of Sassari, Sassari, Italy; 5 Department of Restorative, Preventive and Pediatric Dentistry, University of Bern, Freiburgstrasse 7, 3010, Bern, Switzerland; 6 Präventivzahnmedizin und orale Epidemiologie der Lutz Zürrer Stiftung, Klinik für Zahnerhaltung, Präventiv- und Kinderzahnmedizin, Zahnmedizinische Kliniken (ZMK), Universitär Bern, Freiburgstrasse 7, 3010 Bern, Switzerland; 7 Department of Surgery, Microsurgery and Medicine Sciences, School of Dentistry, University of Sassari, Sassari, Italy; 8 WHO Collaborating Centre for Epidemiology and Community Dentistry, University of Milan, Milan, Italy

**Keywords:** Probiotic, Lactobacillus brevis CD2, Caries, Plaque pH, Salivary mutans streptococci, Children

## Abstract

**Objective:**

The short-term effect (60 days) of *Lactobacillus brevis* CD2 lozenges vs placebo on variables related to caries and gingivitis in type 1 diabetic children was evaluated.

**Material and methods:**

Eight diabetics (4–14 years old) were assigned to two groups (*n* = 34 subjects each), probiotic lozenges and placebo. Stimulated saliva for microbiological analysis and plaque pH were assessed at baseline (*t*_0_), 30 days (*t*_1_), 60 days (*t*_2_) and in the follow-up period (90 days from baseline, *t*_3_). Gingival status was assessed at *t*_0_, *t*_2_ and *t*_3_. Two-way ANOVA assessed differences between groups.

**Results:**

In the probiotic group, *Streptococcus mutans* bacterial density mean scores dropped from 3.11 ± 1.13 at baseline to 1.82 ± 0.72 (*t*_2_) and to 2.06 ± 0.56 (*t*_3_), while in the placebo group, the scores were 3.09 ± 0.8 (*t*_0_), 2.82 ± 0.47 (*t*_2_) and 3.11 ± 0.43 (*t*_3_) (*p* < 0.01). Lowest and maximum pH fall increased in the probiotic group, from 5.37 ± 0.41 at baseline to 5.49 ± 0.24 at *t*_3_ (*p* < 0.01) and from 1.20 ± 0.46 to 0.98 ± 0.29 (*p* = 0.02). Bleeding score decreased significantly in both groups, showing a statistically significant lower bleeding score at *t*_2_ in the probiotic group (25.6%, 95% CI 21.5–32.7 vs 29.5%, 95% CI 25.2–34.9, *p* = 0.02).

**Conclusions:**

*Lactobacillus brevis* CD2 has shown to improve caries-related risk factors and gingival health in diabetic children.

**Clinical relevance:**

*Lactobacillus brevis* CD2 might contribute to improved oral health in type 1 diabetic children.

**Electronic supplementary material:**

The online version of this article (10.1007/s00784-020-03342-0) contains supplementary material, which is available to authorized users.

## Introduction

Diabetes mellitus is a chronic disease resulting from a relative or absolute deficiency of insulin, which affects the metabolism of carbohydrate, protein and fat [1]. Commonly occurring complications are nephropathy, dyslipidaemia, neuropathy, and retinopathy [2]. Sardinia (Italy) together with Finland and Sweden are known to have the highest incidence of type 1 diabetes in the world [1, 3–6].

Diabetic patients do often show high prevalence of gingivitis, periodontal disease and xerostomia, and these comorbidities can be correlated with the disease duration and degree of the metabolic control [7]. Changes in the oral microflora of diabetic subjects in relation to poor glycaemic control may significantly influence the prevalence of gingivitis and caries [7–10]. An association between diabetes and dental caries has been postulated. Unbalanced diabetes (HbA1c < 7.5%-58 mmol/mol) [10–12] is associated with significant cariogenic changes in the oral environment, including less resting and stimulated whole saliva, lower saliva buffering capacity and pH, higher salivary glucose and albumin concentrations, higher proportion of salivary mutans streptococci and yeast [12, 13]. Recently, it was demonstrated that diabetic children in good metabolic control are considered at low caries risk, while those in bad metabolic control showed an oral environment prone to caries development [10].

Different strategies, including the use of probiotic strains, have been suggested in order to prevent gingivitis and caries [14–20]. The major drawback of the use of probiotic for oral health is that the majority of probiotics used are not oral bacteria and so a daily administration is needed to maintain the positive effect [15]. In different fields of oral health care, probiotics have demonstrated a clinical effect on different oral conditions such as halitosis, oral candidiasis and dental caries [17–20]. The use of probiotics in caries prevention leads to the inhibition of the proliferation of cariogenic bacteria (mainly *Streptococcus mutans*) and the reduction of bacteria adherence to the tooth surfaces. The effect of different strains of probiotics has been evaluated, obtaining a reduction in caries incidence, a reduced concentration of *mutans* streptococci and lactobacilli in plaque and saliva, a decreased plaque acidogenicity and a reversal of root caries lesions [11, 16, 18–24].

The hypothesis behind this randomised clinical trial (RCT) was that the administration of probiotic lozenges containing *Lactobacillus brevis* CD2 will be able to modify the oral microflora composition, biofilm acidogenicity and gingival health in children diagnosed at least 2 years before with type 1 diabetes. The null hypothesis was that the probiotic strain would not modify the oral health-related variables.

## Material and methods

### Study design and sample

The study protocol was approved by the Ethical Committee of the University of Sassari [protocol number 133/2014] and conducted according to the principles of the Helsinki Declaration II (ClinicalTrials.gov Identifier: NCT01778699)

The study was carried out in the Dental Clinic of the University of Sassari, School of Dentistry, Sassari (Italy) and lasted from May 2016 to March 2017.

The inclusion criteria were (1) age between 4 and 14 years, (2) type 1 diabetes diagnosed at least 2 years before, (3) good general health except for diabetes and (4) reported to clean the teeth at least twice a day. Exclusion criteria were (1) ongoing oral/dental treatment except for emergency treatment, (2) presence of the oral mucosa diseases, (3) use of fluoride-containing products except for toothpaste within the 14 days from the beginning of the trial, and (4) antibiotic therapy within the past 6 months.

A sample size calculation was performed before the start of the trial using the web-based OpenepiTM platform (http://openinfo.com), considering a difference between the two groups of 5% regarding probiotic effects on oral health in children [16, 17, 25]. The bilateral significance level was set at 95% with a power of 80%. The number of diabetic children needed to be enrolled was fixed in 64. Information on the study aim and design was mailed to 75 parents of children with type I diabetes treated at the Paediatric Clinic of the University of Sassari (Italy), asking the consent for their child participation into the study. Seventy-two diabetic children agreed to participate and 68 were enrolled. HbA1c levels were obtained from their medical charts.

**Fig. 1:**
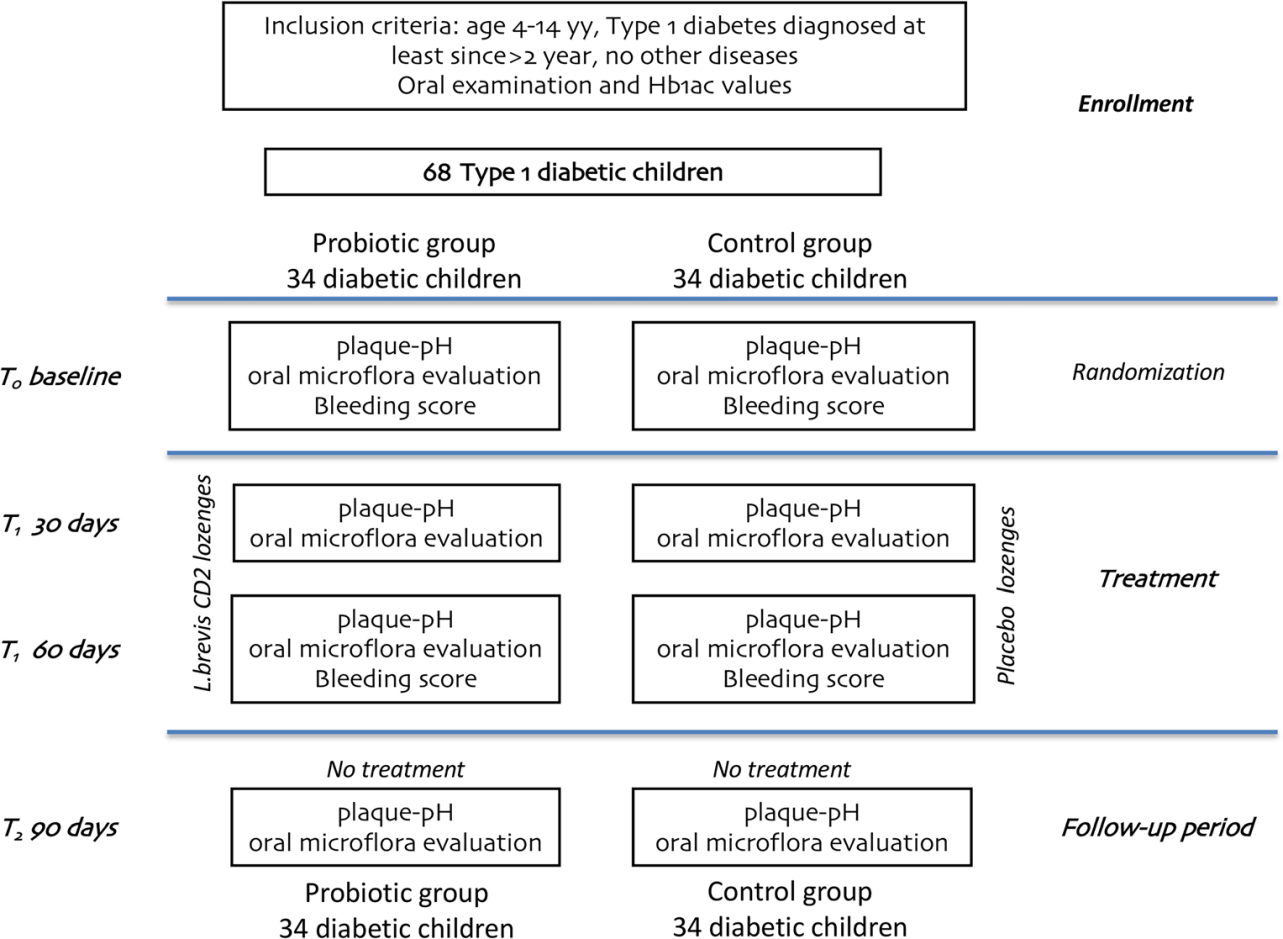
Flow chart of the study

Randomisation was carried out on an individual basis by FC using Excel® 2010 for Mac and two groups of 34 children each were created: (1) a probiotic group, using non-sucrose lozenges containing *Lactobacillus brevis* CD2 and (2) a control group, using non-sucrose lozenges with no active ingredients. The flowchart of the study design is reported in Fig. 1. One week before the start of the experiment, all the subjects began to use a 1400-μg/g AmF toothpaste (Gaba-Colgate, Rome), for daily oral hygiene. A soft toothbrush was likewise provided, and they were asked to avoid any other oral hygiene adjuvant.

### Treatment

The probiotic lozenges (Inersan®, CD Investments srl, Rome, Italy) contained 2,000,000,000 colonies of *Lactobacillus brevis* CD2, sweeteners (mannitol, aspartame, fructose), anticaking agents (talc, silicon dioxide, magnesium stearate) and banana flavouring. The lozenges for the control group contained exactly the same ingredients, except for the *L. brevis* CD2. The two lozenges were identical in weight (1 g), form, colour and packing and they were coded as either “green” or “red”. The code was sealed by an independent monitor and was not broken until the statistical analysis was finalised. Each subject took two lozenges a day, one in the morning and one in the evening, during the whole experimental period (60 days). The compliance and any observed side effects of the products were assessed by means of a questionnaire administered to the participants’ parents at day 60 (*t*_2_). Children were instructed to slowly dissolve the lozenges before swallowing them and to refrain to eat or drink for at least 30 min after exposure.

### Clinical examination, saliva sampling and pH measurement

All subjects were instructed not to brush their teeth or to eat/drink during the hour before the oral examination.

The clinical examination was made under optimal lighting using a mirror and a World-Health-Organization probe to assess caries lesions and gingival conditions. The WHO probe has a coloured band (called the reference marking) located 3.5–5.5 mm from the probe tip. Caries registration was realised at baseline using the International Caries Detection and Assessment System (ICDAS) [25]. No radiographs for caries diagnosis were used [22]. Furthermore, the bleeding on probing score for all teeth was assessed at baseline (*t*_0_), at the end of the treatment (*t*_2_) and in the follow-up period (*t*_3_). Data on their medical condition was also retrieved from their medical charts.

Saliva sample was collected during 5 min of continuously spitting into a test tube after 60 sec of pre-stimulation using paraffin gum [26, 27]. Younger children were instructed on the collection procedure and followed during the test, instructing them repeatedly to spit during the entire procedure time.

Immediately after the saliva sampling, plaque acidogenicity was assessed using the pH indicator strips in 2 maxillary interproximal spaces: (1) between the first and the second right and left primary molars in the younger children or (2) between the 2nd primary and 1st permanent right and left molars in the older children. Measurements were performed before (0 min) and at 2, 5, 10, 15, 20 and 30 min after a mouth rinse with 10% sucrose solution for 1 min. The strips measure a pH value in the range of 4.0–7.0 (Spezialindikator, pH range 4.0–7.0; Merck, Darmstadt, Germany) [27–29]. Each strip was cut into 4 pieces (approx. 2 mm in width) to get a strip that more easily could be inserted into the interproximal space. The strip was held in situ for 10 s after which it was removed, and its colour was compared to the colour index scheme supplied by the manufacturer. The pH was determined to one decimal of the value. For each site, 3 measurements were carried out at each time point [30, 31]. A Stephan curve was generated for each participant at each visit, with three parameters derived from the curve, namely, the lowest pH reached, the maximum pH fall and the AUC below the enamel critical pH (5.5).

### Microbiological analyses

The saliva samples were sent to the Department of Microbiology, University of Sassari for the evaluation of oral microflora [32].

The microbiological analysis was made using the checkerboard DNA-DNA hybridisation method [28]. Whole genomic probes were matched from 9 bacterial strains grouped in primary cariogenic bacteria (*Streptococcus mutans*, *Streptococcus sobrinus*, *Lactobacillus casei* and *Lactobacillus fermentum*) and bacteria known not to be primary associated with caries (*Streptococcus mitis*, *Streptococcus gordoni*, *Lactobacillus salivarius*, *Streptococcus sanguinis* and *Streptococcus salivarius*). Matching the obtained signals with the ones generated by the pooled standard samples, containing a count of 10^5^ and 10^6^ of each bacterial species respectively, an evaluation of the bacterial count was performed in the samples [27].

### Statistical analyses

All the data were input into a spreadsheet (Microsoft Excel1 2011 for Mac, version 14.4.3). Statistical analyses were performed using Stata/SE1 software, version 13.1 for Mac (64-bit Intel). All inputs in the electronic data file were double-checked (data were entered and analysed twice) and then the results were compared.

Caries data were grouped as follows: healthy/caries-free (ICDAS 0), initial (caries in enamel ICDAS 1–2), moderate (caries not cavitated, ICDAS 3–4) and severe (cavitated caries in dentin, ICDAS 5–6). The bleeding score, as the percentage of periodontal sites bleeding on probing, was calculated.

Data from the microbiological analysis was coded on a scale from 0 to 5: 0 = no signal; 1 = a signal density weaker than that of the low standard (< 10^5^ bacteria); 2 = a signal density equal to that of the low standard (= 10^5^ bacteria); 3 = a signal density higher than that of the low standard but lower than that of the high standard (> 10^5^ but < 10^6^ bacteria); 4 = a signal density equal to that of the high standard (= 10^6^ bacteria) and 5 = a signal density higher than that of the high standard (> 10^6^ bacteria).

The mean plaque pH (± standard error) for all subjects measured in the two interproximal sites at the different time points was calculated. The lowest pH value and the maximum pH fall (difference in pH units between baseline and lowest pH) after the sucrose rinse were calculated for each subject.

Comparisons of the different variables were made between the diabetic subjects treated with lozenges containing *Lactobacillus brevis* CD2 and diabetic subjects treated with placebo. All data was analysed univariately to describe the variables and distributions. To avoid the attenuating effect of unequal variability between groups on the value of *t*, a square root transformation was performed when the response variable was a count. One-way analysis of variance (ANOVA) was performed for means comparison between the two groups [33]. For assessment of the difference between being in the intervention group or in the control group, mixed-model analysis for repeated measurements was performed. To achieve comparable conditions between groups, the variables gender (factor), microbiological data and plaque pH (covariate) and Hb1Ac as measured by the TTMC (factor, attempts until success) were also included in the models. Fitting of the models was checked graphically by plotting of the residuals. *p* < 0.05 was considered statistically significant.

## Results

There were no reports of any side effects in the probiotic and in the control groups. Only one child belonging to the probiotic group reported having forgotten a single lozenge intake.

Caries data are reported in Table 1. No statistically significant differences were observed in the two groups: the majority of the subjects were caries-free and a restricted minority had severe caries lesions.

**Table 1:** Caries status (ICDAS index) in diabetic subjects, the children using lozenges with *Lactobacillus brevis* CD2 (probiotic group) and lozenges without probiotic bacteria (control group) after randomization

Caries status	Probiotic group % (*n*)	Control group % (*n*)	*p* value
Caries free (ICDAS 0)	61.9 (21)	55.9 (19)	NS
Initial caries (ICDAS 1-2)	17.6 (6)	23.5 (8)	NS
Moderate caries (ICDAS 3-4)	17.6 (6)	14.7 (5)	NS
Severe caries (ICDAS 5-6)	2.9 (1)	5.9 (2)	NS

Table 2 shows the microbiological results in the two groups recorded at baseline, after 30 (*t*_1_) and 60 days (*t*_2_) of lozenges’ use and 30 days after the cessation of use (day 90, *t*_3_). In the probiotic group, both primary and not primary cariogenic bacteria decreased during the experimental period, except for *Lactobacillus salivarius*, whose concentration did not undergo significant changes; *Streptococcus mutans* concentration showed the greatest reduction dropped from 3.11 ± 1.13 at baseline to 1.82 ± 0.72 at *t*_2_ and to 2.06 ± 0.56 at *t*_3_, respectively (*p* < 0.01). None significant change was noted for the concentration of any bacterial species in the control group during the experimental period. The comparison between groups shows statistically significant differences between at 30, 60 and 90 days. At the end of the treatment period (*t*_2_), all bacterial species were statistically significant different when comparing the two groups, with *Streptococcus mutans* and *Lactobacillus casei* showing the highest differences (1.82 ± 0.72 vs 2.82 ± 0.47 for *S. mutans* and 1.65 ± 0.60 vs 2.22 ± 0.47 for *Lactobacillus casei*, *p* < 0.01 for both). This difference, although reduced, was also recorded at *t*_1_ and *t*_3_ for *Streptococcus mutans*, *Streptococcus sobrinus* and *Streptococcus sanguis*, only at *t*_1_ for *Lactobacillus casei* and *Lactobacillus salivarius* and only at *t*_3_ for *Streptococcus salivarius.*

**Table 2:** Bacterial density scores in the two groups are presented, recorded before (at baseline) and after 30 and 60 days of the lozenges use and 30 days after the cessation of use

Bacteria	Probiotic group					Control group					Comparison between groups		
	Mean *±* SD					Mean *±* SD					*p* value		
Primary cariogenic bacteria													
	Baseline	30 days	60 days	90 days	*p* value	Baseline	30 days	60 days	90 days	p- value	30 days	60 days	90 days
	(*t*_0_)	(*t*_1_)	(*t*_2_)	(*t*_3_)		(*t*_0_)	(*t*_1_)	(*t*_2_)	(*t*_3_)		(*t*_1_)	(*t*_2_)	(*t*_3_)
*S. mutans*	3.11 ± 1.13	2.15 ± 0.65	1.82 ± 0.72	2.06 ± 0.56	< 0.01	3.09 ± 1.08	2.76 ± 0.60	2.82 ± 0.47	3.11 ± 0.43	NS	0.02	< 0.01	0.02
*S. sobrinus*	1.76 ± 0.74	1.50 ± 0.70	1.47 ± 0.66	1.54 ± 0.66	0.03	2.00 ± 0.65	2.20 ± 0.41	2.11 ± 0.32	2.06 ± 0.24	NS	0.01	0.02	0.02
*L. casei*	2.43 ± 1.81	1.76 ± 0.70	1.65 ± 0.60	2.17 ± 0.51	0.02	2.24 ± 1.05	2.18 ± 0.55	2.22 ± 0.47	2.18 ± 0.39	NS	0.03	< 0.01	NS
*L. fermentum*	2.47 ± 0.86	1.91 ± 0.50	1.75 ± 0.49	1.99 ± 0.46	0.04	2.36 ± 0.90	2.18 ± 0.55	2.26 ± 0.45	2.21 ± 0.41	NS	NS	0.03	NS
Not primary cariogenic bacteria													
*S. sanguinis*	2.50 ± 0.86	2.14 ± 0.82	2.05 ± 0.81	2.27 ± 0.71	0.03	2.89 ± 1.12	2.68 ± 0.64	2.53 ± 0.61	2.92 ± 0.46	NS	0.03	0.01	< 0.01
*S. salivarius*	2.54 ± 1.01	2.44 ± 0.69	2.19 ± 0.61	2.37 ± 0.56	0.04	2.61 ± 0.92	2.56 ± 0.50	2.52 ± 0.47	2.63 ± 0.41	NS	NS	0.04	0.04
*S. mitis*	2.26 ± 1.08	1.80 ± 0.75	1.68 ± 0.65	2.02 ± 0.59	0.03	2.26 ± 1.11	2.25 ± 0.66	2.21 ± 0.56	2.22 ± 0.33	NS	0.04	0.01	NS
*S. gordonii*	2.47 ± 0.89	2.17 ± 0.51	1.94 ± 0.50	2.32 ± 0.47	0.02	2.42 ± 0.94	2.41 ± 0.56	2.35 ± 0.49	2.27 ± 0.39	NS	NS	0.01	NS
*L. salivarius*	2.09 ± 0.62	1.85 ± 0.65	1.78 ± 0.72	1.93 ± 0.66	NS	2.05 ± 1.05	2.12 ± 0.62	2.12 ± 0.48	2.09 ± 0.36	NS	0.04	0.04	NS

Table 3 shows the plaque pH measurements expressed as the lowest pH reached and the maximum pH fall in the two groups recorded at *t*_0_, *t*_1_, *t*_2_ and *t*_3_. Both parameters increased significantly in the probiotic group: lowest pH changed from 5.37 ± 0.41 at baseline to 5.49 ± 0.24 at *t*_3_ (*p* < 0.01) and maximum pH fall from 1.20 ± 0.46 to 0.98 ± 0.29 (*p* = 0.02) in the same interval. No significant differences were recorded for both pH parameters in the control group. Regarding comparison between groups, only the lowest pH differed in a statistically significant way at the end of the lozenges’ use, reaching the value of 5.69 ± 0.29 in the probiotic group vs 5.48 ± 0.40 in the control group (*p* = 0.04). Maximum pH fall did not differ between the two groups in any evaluation.

**Table 3:** Plaque pH measurements in the two groups, recorded before (at baseline) and after 30 and 60 days of the lozenges use and 30 days after the cessation of use

	Lowest pH			Maximum pH fall		
	Probiotic group	Control group	*p* value	Probiotic group	Control group	*p* value
Baseline (*t*_0_)	5.37 ± 0.41	5.34 ± 0.50	NS	1.20 ± 0.46	1.18 ± 0.56	NS
30 days (*t*_1_)	5.46 ± 0.37	5.42 ± 0.44	NS	1.10 ± 0.42	1.13 ± 0.51	NS
60 days (*t*_2_)	5.69 ± 0.29	5.48 ± 0.40	0.04	1.02 ± 0.35	1.06 ± 0.45	NS
90 days (*t*_3_)	5.49 ± 0.24	5.43 ± 0.43	NS	0.98 ± 0.29	1.07 ± 0.51	NS
One-way ANOVA *p* value	< 0.01	NS		0.02	NS	

A statistically significant decrease of the bleeding score was recorded in both groups from baseline to the last follow-up examination, even if it was more pronounced in the probiotic group *p* = 0.03 in the control group and *p* < 0.01 in the probiotic group (Table 4). In the comparison between groups, subjects using the probiotic lozenges showed a statistically significant lower bleeding score at the end of the treatment period (*t*_2,_ 60 days) compared to the control group (25.6 vs 29.5, *p* = 0.02). This difference was no more evident 30 days after the cessation of the lozenges’ administration (32.6 vs 31.9).

**Table 4:** Bleeding scores in the two groups, recorded at baseline (*t*_0_), at the end of the lozenges administration (*t*_2_) and 30 days after (*t*_3_)

Bleeding scores	Probiotic group % (95% CI)	Control group % (95% CI)	*p*-value
Baseline (*t*_0_)	34.5 (27.8–42.3)	33.4 (27.0–39.6)	NS
60 days (*t*_2_)	25.6 (21.5–32.7)	29.5 (25.2–34.9)	0.02
90 days (*t*_3_)	32.6 (24.6–32.7)	31.9 (23.8–36.0)	NS
*p* value (one-way ANOVA)	< 0.01	0.03	

The multivariate model showed a statistically significant (*p* < 0.01) association between the probiotic administration (dependent variable), the concentration of primary cariogenic bacteria, the lowest pH, and the bleeding scores. The estimates for these independent variables were comparable, showing there were only small differences between groups (Table 5).

**Table 5:** Multivariate stepwise linear regression analysis: the administration of probiotic vs placebo lozenges as dependent variables

Predictors	Unstandardised Coefficient (B)	*t* statistics	Significance (*p*)	95% confidence interval
Gender	0.264	2.14	0.25	0.46 to 2.47
Hb1Ac	0.256	2.04	0.35	0.44 to 3.02
Bleeding scores	0.682	3.13	< 0.01	0.35 to 0.84
Minimum pH	0.324	3.10	< 0.01	0.25 to 0.86
Primary cariogenic bacteria	0.335	2.75	< 0.01	0.13 to 0.53
Not primary cariogenic bacteria	− 0.259	2.01	0.18	− 0.25 to 1.04

## Discussion

The goal of this RCT was to evaluate the effect of *Lactobacillus brevis* CD2 administered through lozenges compared to a placebo in a sample of diabetic children diagnosed at least 2 years before. Results showed that the 60-day administration of the probiotic strain produces a significant reduction of cariogenic microorganisms compared to the placebo. A statistically significant, but meager in absolute value, difference between groups was also noted in the plaque pH (lowest pH value) and in the bleeding score at the end of the administration period.

Although a lower prevalence of caries lesions was recorded in diabetic group than in non-diabetic population at the onset of the disease, a higher caries prevalence has previously been found in diabetic subjects with bad metabolic control compared to diabetic subjects with good metabolic control [10, 12]. These data might be linked to a more cariogenic microbial flora and consequent lower plaque pH values due to the higher sugared snack and beverage intake as well as leakage of glucose from the gingival crevicular flow in the non-stabilized metabolic subjects [7, 9, 10]. Cariogenic bacteria reached a higher saliva concentration (*Streptococcus mutans* and *Streptococcus sobrinus*) in diabetic children compared to non-diabetic children, and this concentration was even higher when a comparison between diabetic children in bad metabolic control vs non-diabetic children was performed [10]. Salivary mutans streptococci concentration was statistically significant associated to caries experience in diabetic children [12].

The main result of the present RCT was that the use of the *Lactobacillus brevis* CD2 lozenges statistically significantly affects the salivary concentration of the considered primary cariogenic bacteria compared to placebo use. The concentration of *Streptococcus mutans* and *Streptococcus sobrinus* decreased during the use of the probiotic lozenge, reaching the highest decrement at the end of the probiotic administration period. Noteworthy, 30 days after the cessation of probiotic use, the concentration was still statistically significant lower compared to control group. Scientific literature reports heterogeneous results on the effect of probiotics on *Lactobacillus* spp. [18]. In the present RCT, the concentration of *Lactobacillus* spp. i.e*. Lactobacillus casei* and *Lactobacillus fermentum* decreased during the experimental period, but it increased again after the cessation of probiotic’s use, underlining the importance of the continuous administration of the probiotic to produce a lasting effect, as widely reported in literature [17–24].

Plaque pH parameters are used for the evaluation of food cariogenicity and/or individual caries risk assessment [28–31]. A more acidogenic oral environment has previously been demonstrated in diabetic children compared to non-diabetics [10].

It has previously been demonstrated that, *Lactobacillus brevis* CD2 lead to a lower plaque acidogenicity compared to a placebo in high caries-risk children [20]. A certain number of probiotics showed to be able to produce bacteriocins or similar substances and usually the effect is pH dependent. *Lactobacillus brevis* CD2 is a functional *Lactobacillus* strain with peculiar biochemical features, essentially related to the activity of arginine deiminase [31]. This enzyme catalyses arginine and affects the biosynthesis of polyamines (putrescine, spermidine, and spermine). Findings of the present study confirm previous results, showing that both the lowest pH and the maximum pH fall were significantly affected by the probiotic use. Nevertheless, the comparison between the two groups showed a statistically significant difference at the end of the administration period (*t*_2_) for the lowest pH only, underlining the slight pH modification produced by the treatment. It is possible to speculate that the duration of the intervention is directly linked to the modification of plaque pH [11, 19, 20]. The pH strip method used showed to be comparable with the micro-touch method [30, 31] taking into consideration that both methods measure pH only on the plaque surface and in the inter-proximal dental space rather than in the depth of the dental plaque.

The change in the concentration of primary cariogenic bacteria may explain the change in the plaque pH values. The change of the pH values recorded, even if moderate, is adequate to reduce the caries risk and it is similar to the modification reported in literature [15, 19, 20]. Otherwise, some results about plaque pH would require more investigation i.e*.* the maximum pH fall at *t*_1_ and *t*_2_ recorded in the control group. One hypothesis could be that children in the control group respond to a placebo effect as a slight improvement in plaque pH is found as they were using a lozenge resulting in increased salivary secretion rate and thereby positive salivary effects during the study period.

An important parameter associated to gingival health, the bleeding score, was also evaluated. A reduction in gingival bleeding was found during the trial in both groups, but a statistically significant difference between groups was found only at the end of the probiotic administration. The improvement in the bleeding score in the control group might be related to the behaviour modification of the subjects enrolled into a trial, producing a bias in the study results and reducing the differences between groups. An anti-inflammatory effect of *Lactobacillus brevis* CD2 administered to a group of patients with chronic periodontitis was reported and related to the capacity of the probiotic to prevent the production of nitric oxide and, consequently, the release of PGE2 and the activation of MMPs induced by the nitric oxide [34].

The optimal dose of probiotic strains for caries prevention is yet to be clarified. From an analysis of the literature, a huge interval ranged between 10^7^ and 10^9^ bacteria is reported [35, 36]. This RCT used a quite high dose (four billion of colonies/die), which is high enough to expect some kind of effect. It seems likely that probiotic lactobacilli, like *Lactobacillus brevis* CD2, might have played an antagonistic effect on cariogenic bacteria i.e. *Streptococcus mutans* and *sobrinus* in plaque biofilm.

Some limits of the study design need to be underlined. First of all, the characteristics of the study population, children affected by type I diabetes, do not allow to generalise the results to the general diabetic population, including adults, taking into consideration that the majority of studies were carried out on adults. Although claimed that all subjects followed the instructions given, it may still be questioned whether this holds for children at all ages. Furthermore, no analyses have been made in relation to years since disease onset, but it cannot be excluded that this is a factor that may influence the findings.

Another limitation might be the variation of plaque pH and bleeding scores in the control group leading to a minimisation of the differences between groups. This might be linked to a “trial-effect bias” as all the subjects received oral hygiene instruction at the enrolment.

Although several studies were carried out on the caries preventive effect of probiotic [37, 38], this study holds almost unique characteristics like the type of the subjects enrolled (type 1 diabetics) and the age of subjects (4–14 years of age), as well as the probiotic used, known to play an important role in oral health. The administration of probiotics with these potentially beneficial properties may be a viable approach to maintain or restore a healthy balance in the microbiota. It has recently been shown that type 1 diabetes at early age debut may have a large impact on general health, including cardiovascular diseases and even mortality [4]. It is believed that this, due to the disease complexity, holds also for oral health.

In conclusion, the 60-day administration of the probiotic strain *Lactobacillus brevis* CD2 through lozenges is able to have a positive effect on important variables related to oral health in diabetic children even if the strength of the effect might be questionable. A reduction of cariogenic microorganisms’ concentration, plaque pH scores and bleeding on probing was recorded. This study provides evidence in favour of the use of *Lactobacillus brevis* CD2 as a promising functional food option to improve oral health in diabetics. Probiotics may be considered a promising adjunct to the current caries management procedures such as fluoridation and modification of dietary sugar intake.

## Electronic supplementary material

ESM 1 (XLSX 185 kb)
